# Root environment is a key determinant of fungal entomopathogen endophytism following seed treatment in the common bean, *Phaseolus vulgaris*

**DOI:** 10.1016/j.biocontrol.2016.09.001

**Published:** 2018-01

**Authors:** Soroush Parsa, Viviana Ortiz, María I. Gómez-Jiménez, Matthew Kramer, Fernando E. Vega

**Affiliations:** a Centro Internacional de Agricultura Tropical (CIAT), Apartado Aéreo 6713, Cali, Colombia; b Statistics Group, United States Department of Agriculture, Agricultural Research Service, Beltsville, MD 20705, USA; c Sustainable Perennial Crops Laboratory, United States Department of Agriculture, Agricultural Research Service, Beltsville, MD 20705, USA

**Keywords:** *Beauveria*, Biological control, Endophytes, Fungi, *Metarhizium*, Seed-borne

## Abstract

The common bean is the most important food legume in the world. We examined the potential of the fungal entomopathogens *Beauveria bassiana* and *Metarhizium anisopliae* applied as seed treatments for their endophytic establishment in the common bean. Endophytic colonization in sterile sand:peat averaged ca. 40% higher for fungus treatments and ca. six times higher for volunteer fungi (other fungal endophytes naturally occurring in our samples), relative to sterile vermiculite. Colonization by *B. bassiana* and *M. anisopliae* was least variable in sterile vermiculite and most variable in sterile soil:sand:peat. The impact of soil sterilization on endophytic colonization was assessed in a separate experiment using six different field-collected soils. Soil sterilization was the variable with the largest impact on colonization (70.8% of its total variance), while the fungal isolate used to inoculate seeds explained 8.4% of the variance. Under natural microbial soil conditions experienced by common bean farmers, seed inoculations with *B. bassiana* and *M. anisopliae* are unlikely to yield predictable levels of endophytic colonization.

## Highlights

• *Beauveria bassiana* and *M. anisopliae* applied as seed treatment in common beans.
• Both fungal entomopathogens become endophytic, with low colonization levels (<30%).
• There was high variability in endophytic colonization.
• Endophytism influenced by status of soil substrate used (sterile or non-sterile).

## 1 Introduction

Living inconspicuously within plant tissues, fungal endophytes play important roles in plant community ecology (Carroll, 1988, Hyde and Soytong, 2008, Omacini et al., 2001, Rodriguez et al., 2009, Saikkonen et al., 1998, Wani et al., 2015), and can have negative effects on insect pests and plant pathogens (Backman and Sikora, 2008, Giménez et al., 2007, Gond et al., 2010) as well as positive effects on plant growth (Behie et al., 2012, Kabaluk and Ericsson, 2007, Sasan and Bidochka, 2012). Consequently, there is widespread interest in learning how to use fungal endophytes with insect pathogenic capabilities (i.e., entomopathogenic fungal endophytes) to enhance plant tolerance to insect pests (Parsa et al., 2013, Vega et al., 2008, Vega et al., 2009). Notable among these fungi are species of *Beauveria* and *Metarhizium*, which are globally distributed and commercially available as biological control agents (Lacey et al., 2001, Lacey et al., 2015). Our objective was to test the potential of these fungi applied as seed treatments for their endophytic establishment in the common bean, *Phaseolus vulgaris*.

Seed treatments with biological control agents provide an environmentally friendly alternative to the use of chemical inputs (Bennett, 1998, Taylor and Harman, 1990). Among them, treatments relying on fungi in the genus *Trichoderma* are particularly well studied (Taylor and Harman, 1990) and have shown significant potential to suppress plant disease and stimulate plant growth (Harman et al., 2004, Mastouri et al., 2010). Seed treatments with the objective of establishing fungal entomopathogens as endophytes, on the other hand, are just beginning to be explored, with interesting experimental results for the management of insect pests (Akello and Sikora, 2012, Akutse et al., 2013, Biswas et al., 2013, Castillo Lopez and Sword, 2015, Castillo Lopez et al., 2014, Cherry et al., 2004, Kabaluk and Ericsson, 2007, Keyser et al., 2014, Mutune et al., 2016, Powell et al., 2009, Quesada-Moraga et al., 2009). Still, very little is known about the relative contributions of genetic factors (i.e., plant and fungal genotype) and environmental factors (e.g., soil organic matter, soil microflora), to the effectiveness of seed treatments with fungal entomopathogens. We addressed this imperative by screening multiple isolates/strains of *Beauveria bassiana sensu lato* (Balsamo-Crivelli) Vuillemin and *Metarhizium anisopliae* (Metchnikoff) Sorokin *sensu lato* (Ascomycota: Hypocreales) under various soil conditions in seed treatments intended to establish the fungal entomopathogens as endophytes in the common bean. An important reason for focusing on seeds is that they are more likely to be adopted by growers because they are easier to use than the other endophyte-related treatments involving foliar sprays, soil drenches, or stem injections involving fungal entomopathogens.

The common bean is the most important food legume in the world (Broughton et al., 2003). Grown in over 12 million hectares, it feeds more than 500 million people in Latin America and Africa alone (Schwartz and Corrales, 1989). This crop is significantly constrained by more than 400 insect pests and 200 plant pathogens, whose attack is thought to be the most limiting bean production factor across regions (Allen et al., 1996, Schwartz and Corrales, 1989). In part due to their impact, bean yields in developing countries average ca. 650 kg ha^−1^, roughly a third of the yield achieved in the US and Canada (Singh, 1999). Developing a seed treatment that can protect the crop from pest and disease attack is therefore well justified.

Parsa et al. (2013) demonstrated that the common bean can be readily colonized by fungal entomopathogens following foliar spray or soil drench inoculations, and that dozens of fungal endophytes can be recovered from germinated seeds (Parsa et al., 2016). Similarly, Gurulingappa et al. (2010) reported establishment of fungal entomopathogens in common bean after leaf spraying. Though successful at an experimental scale, these inoculation methods would require hundreds of liters of concentrated fungal entomopathogen inoculum per hectare, making them costly and impractical for field level applications. A seed inoculation method could offer farmers a cost effective and easy to implement alternative to establish fungal entomopathogens as protective endophytes in the common bean. Akutse et al. (2013) and Mutune et al. (2016) used seed soaking to inoculate *P. vulgaris* with fungal entomopathogens and subsequently examined the effects on *Liriomyza huidobrensis* and *Ophiomyia phaseoli* (Diptera: Agromyzidae), respectively.

## 2 Materials and methods

### 2.1 Fungal inoculum

Eleven isolates/strains each of *B. bassiana* and *M. anisopliae* were obtained from commercial products available in Colombia and from the collections of entomopathogenic fungal cultures maintained by the International Center for Tropical Agriculture (CIAT), the Colombian Corporation for Agricultural Research (CORPOICA), and the Colombian Oil Palm Research Center (Cenipalma). Monosporic cultures were prepared following procedures described by Parsa et al. (2013). Briefly, the cultures were grown on potato dextrose agar (PDA; Difco Laboratories, Becton Dickinson, Sparks, MD) in 100 × 15 mm Petri dishes and incubated in the dark at 26 ± 2 °C for 14–18 d, after which conidia were harvested by scraping the surface of the plate with a sterile spatula and suspending the fungal mass in sterile 0.1% Triton X-100 (Sigma Aldrich, St Louis, MO). The suspension was mixed and then filtered through sterile cheesecloth to remove mycelium and agar debris. The conidial concentration was determined using an improved Neubauer hemacytometer (Hausser Scientific, Horsham, PA) and adjusted to 10^8^ conidia m L^−1^ in maize oil (Industrias del Maíz S.A., Cali, Colombia), which we used as a sticker to adhere conidia to the seed surface (Jackson et al., 2010). A germination test to evaluate conidial viability was carried out by plating a 100 μl aliquot of a 10^−4^ serial dilution of the 10^8^ conidia m L^−1^ conidial stock suspension on 2.5% water agar (Sigma Aldrich, St Louis, MO). Plates were incubated in the dark at 26 ± 2 °C for 24 h after which three groups of 100 randomly selected conidia were evaluated to assess percent conidial germination. Conidia were deemed germinated when the length of the germ tube exceeded half the diameter of the conidia.

### 2.2 Seed sterilization

*Phaseolus vulgaris* cv. Calima seeds were obtained from plants grown at CIAT. Prior to their inoculation, they were surface-sterilized by immersion in 0.1% Triton X-100 for 2 min, 0.5% sodium hypochlorite for 2 min, and 70% ethanol for 2 min, followed by three rinses with sterile distilled water. To assess the effectiveness of the sterilization method, three randomly selected seeds from 15 or 45 seeds used in the screening and full experiments, respectively (see “Seed inoculation,” below), were individually pressed onto ¾-strength PDA media plates (Schulz et al., 1998, Greenfield et al., 2016). Plates were incubated at 26 ± 2 °C in darkness and were examined after 10 d. The disinfection was deemed successful when no fungal growth was observed on the PDA plate. If fungal growth was observed, the sample was discarded and not considered for endophyte isolation.

### 2.3 Seed inoculation

Groups of 15 seeds (see “Screening experiments,” below) or 45 seeds (see “Full experiments,” below) were transferred into sterile 100 x 20 mm Petri dishes and air dried in a laminar flow hood for approximately 1 h before inoculation. Surface sterilized seeds were immersed in 1 × 10^8^ mL^−1^ conidial suspensions for 2 min. Immersion was done by adding approximately 15 ml of the suspension into the Petri dishes containing the seeds. Prior to the inoculation, the conidial suspension was manually shaken for 30 s. Control sterilized seeds were prepared by immersion in 0.1% Triton X-100 and maize oil in the same proportion as treatments. Each inoculated seed was sown into the corresponding substrate in a 50 cm^3^ germination tray cell (PlastiKa Asociados Ltda., Bogotá, Colombia). Trays were arranged randomly in plant growth chambers at 25 ± 2 °C, 47% relative humidity (RH) and 12:12 photoperiod (10,000 lx). Plants were watered daily with ca. 8 ml of sterile tap water starting the third day after inoculation (dai) until 16 dai, then every other day with 50 ml of tap water until 30 dai. Plants were then watered with 100 ml of tap water every other day for the remainder of the experiment.

### 2.4 Potting substrate sterilization

To validate the effectiveness of the potting substrate sterilization methods (discussed below) in eliminating fungal spores, 1 cm^3^ of each sterilized potting substrate sample was suspended in 9 ml of sterile 0.1% Triton X-100 and vortexed for 3 min, followed by plating 100 μl of 10^−1^ and 10^−2^ dilutions of this suspension onto ¾-strength PDA and PDA media plates amended with penicillin (100 mg L^−1^), streptomycin (200 mg L^−1^) and tetracycline (50 mg L^−1^). Antibiotics were filter-sterilized through a 0.2 μm filter Nalgene syringe filters with 25-mm surfactant-free cellulose acetate (Nalge Nunc International Rochester, NY). Plates were incubated at 26 ± 2 °C in darkness and were checked after 10 d for any fungal growth. No fungal growth was observed in any of the samples.

### 2.5 Experimental design

Five separate experiments were conducted as completely randomized designs with factors varying among experiments. Three experiments (see “Screening experiments,” below) were run to evaluate the endophytic potential of 11 *B. bassiana* isolates and 11 *M. anisopliae* isolates. The fourth experiment (see “Full experiments,” below) evaluated the effects of substrate and dai on common bean plant colonization with two selected isolates of each fungal species, and with four replicates over time. The fifth experiment (see “Sterile and non-sterile soils experiment,” below) evaluated the effect of soil sterilization on endophytic colonization achieved by seed treatments with two fungal entomopathogens. For this experiment, soil samples were collected from six bean production fields in Colombia (see “Sterile and non-sterile soils experiment,” below*)*. This experiment was conducted twice.

### 2.6 Screening experiments

Three separate screening experiments were conducted in a growth chamber to evaluate the ability of the 22 fungal entomopathogen isolates/strains to endophytically colonize common bean plants. The first experiment used six *M. anisopliae* isolates/strains: CIAT 001, CIAT 014A, CIAT 042, CIAT 053, BioMa® (Bio-Protección, Chinchiná, Colombia), and Metarhiplant® WP (Sanoplant, Palmira, Colombia). The second experiment consisted of six *B. bassiana* isolates/strains: CIAT 359, CIAT 405, Bovetrópico® WP (Soluciones Microbianas del Trópico Ltda., Chinchiná, Colombia), Micosis® WP (Bio-Protección, Chinchiná, Colombia), Beauveriplant® WP (Sanoplant, Palmira, Colombia), and Mycotrol® O (LAM International, Butte, MT, USA). The third experiment used five isolates each of *B. bassiana* (CpBb0417, CpBb0420, Bv012, Bv036, and Bv047) or *M. anisopliae* (CpMa1105, CpMa1107 CpMa1206, Mt008, and Mt009). Screening experiments were designed to eliminate isolates that showed no potential on a first experimental round. Based on results from screening experiments, the two best *Beauveria* and *Metarhizium* isolates were selected for full experiments, which were repeated four times (see below).

Vermiculite (Fumitoro Ltda., Bogotá Colombia) with particle size <0.7 mm was used as a potting substrate for all screening experiments. Vermiculite was sterilized in an autoclave for 20 min at 121 °C using 800 g (0.0012 m^3^) for each sterilization batch. After sterilization, vermiculite was cooled for at least 72 h and moistened with 1300 ml of sterile tap water prior to filling the trays and planting the seeds. Each treatment tray held five bean seeds. Evaluations for endophytic colonization were conducted 8 and 14 dai.

### 2.7 Evaluation for endophyte colonization

Germination trays containing the seedlings were transferred from the growth chambers to the laboratory, where the plants were carefully uprooted. Individual plants were washed under running tap water for approximately 1 min and secondary roots were carefully removed manually, leaving only the taproot. Each plant was divided into two sections, the above ground part and the below ground part. The latter was obtained by cutting approximately 3 cm above the root crown and therefore includes 3 cm of stem. Both plant sections were introduced into a Fisherbrand™ 50 ml polypropylene centrifuge tube (Fisher Scientific, Pittsburgh, PA) to be bulk surface-sterilized following the Greenfield et al. (2015) methodology. The plant sections introduced into the centrifuge tube were agitated for 2 min at 250 ppm in 0.05% Triton X-100 in an Innova 2000 Platform Shaker (New Brunswick Scientific Co. Inc., Edison, NJ). The tube was then transferred to a laminar flow cabinet for sequentially immersion in 0.5% sodium hypochlorite (diluted in 0.05% Triton X-100) for 2 min, followed by 70% ethanol for 1 min and then rinsed in sterile distilled water for three cycles of 15 s each. The disinfected plant sections were removed from the tube and dried on sterile towel paper in a laminar flow hood. To determine the effectiveness of the sterilization process, the section with the below ground part of each plant was gently pressed onto ¾-strength PDA media plates (Schulz et al., 1998) and incubated at 26 ± 2 °C for at least 14 d. Plant sections were then dissected into 12 fragments (see Fig. 1 in Parsa et al., 2016) averaging 2 × 3 mm^2^. The six root fragments were plated separately from the leaf and stem fragments on two 60 mm diameter Petri dishes containing ¾-strength PDA media plates amended with penicillin (100 mg L^−1^) streptomycin (200 mg L^−1^) and tetracycline (50 mg L^−1^). Antibiotics were filter-sterilized as described above. The plates were incubated at 26 ± 2 °C in darkness, and monitored for the presence of *B. bassiana* and *M. anisopliae* for up to 14 d. Presence of fungal endophytes other than *B. bassiana* or *M. anisopliae* was also recorded.

**Fig. 1:**
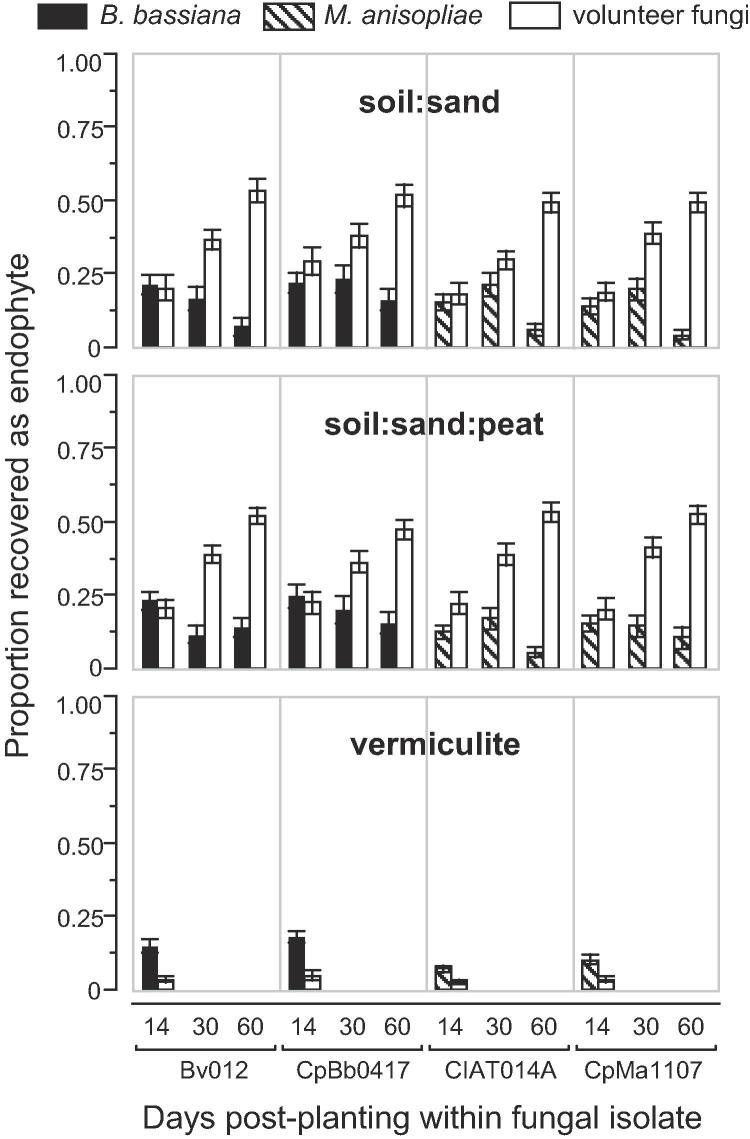
Endophytic colonization of bean seedlings resulting from seed inoculations with fungal entomopathogens in three sterile potting substrates.

### 2.8 Full experiments

Based on the highest levels of endophytic colonization in the screening experiments (data not shown) two *B. bassiana* (Bv012, CpBb0417) and two *M. anisopliae* isolates (CIAT 014A, CpMa1107) were selected. The isolates were used to evaluate the effect of three sterile potting substrates on endophytic colonization of common bean seedlings resulting from seed inoculations (as previously described).

The three sterilized potting substrates were: a mixture of soil:sand (3:1); a mixture of soil:sand:peat (3:1:1); and vermiculite. The soil used was a silt loam soil (ca. 18% clay, 10% sand, and 72% silt) with an organic matter content of 32 g kg^−1^, and was collected from a CIAT experimental field. The soil was mixed with sand (3:1), resulting in an additional 25% sand. The soil:sand:peat had an additional 20% peat (Pindstrup Plus Orange, Sia Pindstrup Latvia, Riga, Latvia). The substrates were sterilized in an autoclave under the same conditions as in the screening experiments. Each fungal entomopathogen-inoculated seed was sown into one of the three substrates in a 50 cm^3^ germination tray cell (PlastiKa Asociados Ltda., Bogotá, Colombia). Trays were randomly placed in growth chambers at 25 ± 2 °C, 47% RH and 12:12 photoperiod (10,000 lx). The plants were grown for 16 d before being individually transplanted into one liter (10 cm diameter × 12 cm height) disinfected pots filled with the same substrate, moistened with 50 ml of tap water before transplantation. The substrate used for transplanting was steam-sterilized at 82 °C for 6 h in 1 m^3^ batches in a media steamer cart using a steam generator. The plants were maintained in the plant growth chambers under the same conditions as before transplantation.

Endophyte colonization evaluation was conducted at 14 (primary leaves fully open; V2), 30 (first flower opens; R6), and 60 (initiating pod fill stage; R8) dai, based on the developmental growth stages proposed by CIAT (Fernández et al., 1986). Plants growing in vermiculite were only tested 14 dai. At each evaluation time, 10 randomly selected plants per substrate per isolate were used and plant parts (leaf, stem, and roots) were evaluated as previously described with the exception that at 30 and 60 dai the sampling location of the leaf and stem sections changed. At 30 dai, each plant was divided into above ground and below ground parts, as previously described. From the above ground part two different sections were taken: the central leaflet of the first trifoliate leaf, trimmed in a square as described by Parsa et al. (2013), and one section of stem, cut 3 cm above and below the node of the first trifoliate leaf. The two sections taken from the above ground part, and the below ground part were disinfected in two separate 50 ml polypropylene centrifuge tubes using the Greenfield et al. (2015) methodology. Disinfected plant sections were dissected into 12 fragments averaging 2 × 3 mm. Two leaf fragments were cut from the middle section of the trimmed leaflet and two stem fragments were cut within 1 cm above and below the node. From the below ground part, two stem fragments were taken from approximately 1 cm above the root crown. The six root fragments were cut as previously described (see Fig. 1 in Parsa et al., 2016). At 60 dai, plants were evaluated as described for 30 dai, except that the second trifoliate leaf was used as reference.

The experiment was replicated four times. At the time of evaluating the second repetition, the colonization by *B. bassiana* and *M. anisopliae* had dropped severely. We suspected a problem with sterilization of the substrate used for transplanting and decided to change the substrate sterilization method in the remaining two replicates. Therefore, substrates used for transplantation were steam-sterilized for 6 h with two cycles using an SST-60 electric soil sterilizer (Pro-Grow Supply Corp., Brookfield, WI) at 82 °C. The total volume of the sterilizer is 0.4 m^3^ (½ yd^3^) and is equally divided into five compartments. Four of the five compartments were filled with soil:sand and were moistened with 32 L of tap water before initiating the sterilization process. The fifth compartment was filled with peat moistened with 12 L of tap water before initiating the sterilization process. Substrates were cooled for 72 h before the second sterilization cycle. Treatments and procedures were the same as described for the two previous replicates.

### 2.9 Sterile and non-sterile soils experiment

A fifth experiment was conducted using six soils and seed inoculated with *B. bassiana* (Mycotrol® O) or *M. anisopliae* (CIAT014A). Mycotrol® O was selected due to its commercial availability in many countries. Five soils were collected from localities where common beans were being grown (Colombian Department in parenthesis): Colón (2137 masl; Putumayo), Gama (2303 masl; Cundinamarca), San Gil (1625 masl; Santander), Río de Oro (1398 masl; Cesar), Dabeiba (1086 masl; Antioquia). The sixth soil was collected at the same CIAT experimental field in Palmira (1000 masl; Valle del Cauca) used for the “full experiments” (see above). For collecting the soils, one soil sample (4 kg from the top 10 cm) was randomly collected. Each soil was tested with and without sterilization. The sterilization treatment for each soil (0.0015 m^3^; 1 kg) consisted of three cycles in an autoclave at 121 °C for 20 min. Between the first and second cycle, a period of 24 h was allowed to pass, and between the second and third cycle, 48 h. Colonization data were collected 14 dai using the same protocol outlined for the screening experiments.

### 2.10 Statistical analysis

We fit mixed models using the R package, lme4 (Bates et al., 2015), to a dataset consisting of the four full experiments (ST10, 11, 13, 14) that were run on different dates. Subsets created from the three substrates were individually modeled. A variance decomposition of the independent variables was conducted by treating all independent variables as random effects. Two analyses were done, both using binomial dependent variables. For the first, we used the count of plant pieces infected by the treatment fungus; for the second, whether or not any piece of the plant was infected (binary). One of the independent variables was the count of plant pieces infected by a fungus other than the treatment fungus, for the variance decomposition we converted this count into a categorical factor with four levels based on quartiles. For the vermiculite substrate, samples were taken only on day 14; there are no estimates for sampling day or its interaction with other independent variables.

## 3 Results

In preliminary analyses for each experiment, we found a clear ordering of the main effects of fungus and media. However, this ordering differed greatly among experiments, and we found no covariate or other factor to explain why. Thus, rather than focus on reporting main treatment effect differences averaged over all experiments (mean responses), which makes little sense given the large experiment-to-experiment variation, we found it better to focus on which variables explained the largest proportions of the variance, through a variance decomposition approach. We found that using this approach for each planting media separately provided the most interpretable results, since this decomposition required only second order interactions to produce models without over-dispersion.

Neither *B. bassiana* nor *M. anisopliae* were recovered as endophytes in any plants that were not inoculated with them (i.e., controls). Preliminary screening experiments in vermiculite with 22 fungal isolates suggested that *B. bassiana* is generally more successful at colonizing bean seedlings than *M. anisopliae*, with the top seven isolates belonging to the former species (data not shown). When considering ease of cultivation in addition to colonization success, the screening experiments identified *B. bassiana* isolates CpBb0417 and Bv012 and *M. anisopliae* isolates CpMa1107 and CIAT014A as potential candidates for seed inoculations. However, within plant colonization was never high, averaging 27 ± 4.4%, 21 ± 4.4%, 10 ± 0.5%, and 6 ± 2.1% for plants inoculated with CpBb0417, Bv012, CpMa1107, and CIAT014A, respectively.

Four subsequent experiments tested the effect of growth medium and sampling day on endophytic colonization by our treatment fungi and any other fungal endophytes naturally-occurring in our samples (Fig. 1). We refer to the latter group of fungi as “volunteer” endophytes, borrowing the term from its comparable use in agriculture to denote plants growing without deliberate human intervention. All else equal, endophytic colonization in sand and peat averaged ca. 40% higher for treatment fungi and ca. six times higher for volunteer fungi, relative to vermiculite (Fig. 1). Over time, mean colonization in soil:sand and soil:sand:peat followed different trajectories for treatment versus volunteer endophytes, with a general tendency of the former to decrease as the latter increased (Fig. 1). Mean colonization varied greatly across the four experimental replicates, with the highest colonization by treatment fungi achieved in the first experiment, where colonization by volunteer fungi was the lowest. Combining all data, we found a negative correlation of −0.30 (p < 0.001) between treatment endophytes (which decreased over time) and volunteer endophytes (which increased over time).

**Fig. 2:**
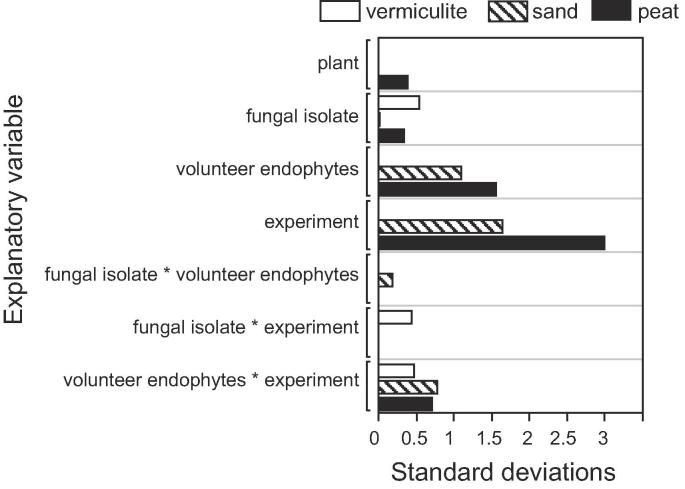
Sources of variation (in units of standard deviation) in endophytic colonization resulting from seed inoculations with fungal entomopathogens in three potting substrates: vermiculite; a mixture of soil:sand (3:1), identified as “sand”; and a mixture of soil:sand:peat (3:1:1) identified as “peat.”.

Colonization by treatment endophytes was least variable in vermiculite and most variable in soil:sand:peat (Fig. 2). Evaluated 14 days after inoculation, variance estimates for colonization by fungal isolate was estimated at 0.7, 4.5 and 12.2 in vermiculite, soil:sand, and soil:sand:peat, respectively (Fig. 2). Note that Fig. 2 is in units of standard deviations (square root of the variance), which better scales the estimates for visual comparisons. Variances, however, are additive, so that percentages of total variance can be calculated.

As a percentage of the total variance for each planting medium (ignoring binomial sampling error), the fungal isolate used to inoculate seeds explained 41.4% of the total variance in vermiculite, but less than 1% of the total variance in soil:sand and soil:sand:peat (Fig. 2). This is a result, in the latter two media, of non-treatment factors, experiment-to experiment-variability, growth of volunteer endophytes, and their interactions with other factors, explaining more than 98% of the total variance (Fig. 2). The variance directly attributable to volunteer endophytes was negligible for vermiculite, but accounted for 26.4% and 19.9% of the variance observed in soil:sand and soil:sand:peat, respectively (Fig. 2). However, for vermiculite, the variance component estimating the interaction between experiment and volunteer endophyte was 31.7%. In sum, the non-treatment factors (those that could not be controlled) in soil:sand and soil:sand:peat had considerably more influence on results than they did for vermiculite. Given the disparate variance decompositions across media, it follows that our screening experiments in vermiculite were unsuitable to assess the endophytic potential of a fungal isolate in the other growth media.

**Fig. 3:**
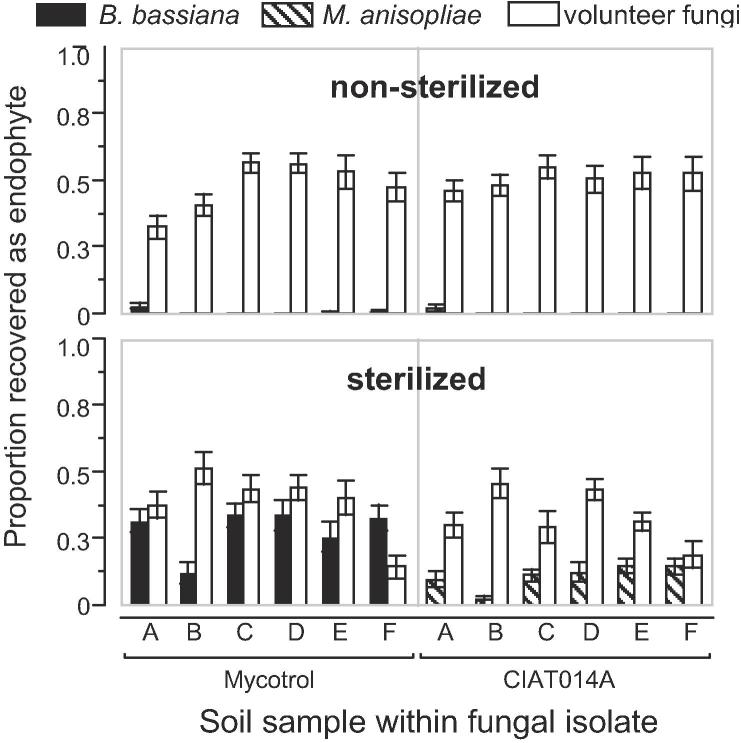
Endophytic colonization of common bean seedlings resulting from seed inoculations with *B. bassiana* (Mycotrol® O) or *M. anisopliae* (CIAT014A). Plants were evaluated 14 days after sowing the treated seeds in sterile or non-sterile soil samples collected in common bean plantations in six Colombian departments (in parenthesis): A: Colón (Putumayo); B: Gama (Cundinamarca); C: San Gil (Santander); D: Río de Oro (Cesar); E: Dabeiba (Antioquia); F: Palmira (Valle del Cauca).

**Fig. 4:**
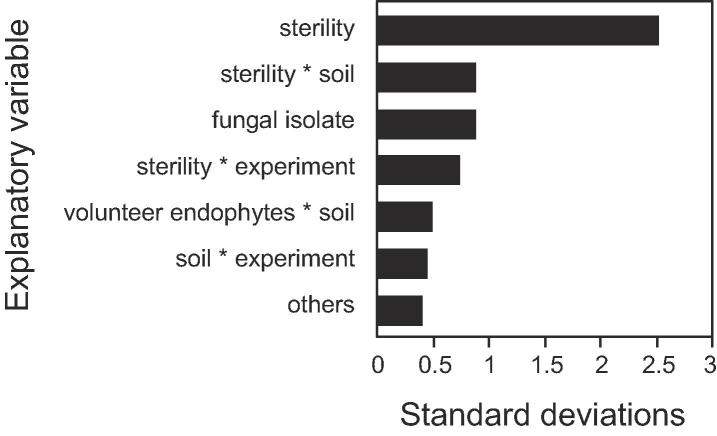
Sources of variation (in units of standard deviation) in endophytic colonization resulting from seed inoculations with fungal entomopathogens. Plants were evaluated 14 days after sowing the treated seeds in soil samples that were either sterilized or not.

We hypothesized this difference, especially for experiment-to-experiment variability, was partly owed to the ease of achieving sterilization in vermiculite relative to media with greater microbial diversity and abundance. Our final experiment was designed to test the impact of soil sterilization, using six different field-collected soils, on endophytic colonization by *B. bassiana* (Mycotrol® O) and *M. anisopliae* (CIAT014A) (Fig. 3). As suspected, soil sterilization was the variable with the largest impact on the colonization achieved by our treatment fungi (Fig. 4), accounting for 70.8% of its total variance (Fig. 4). By contrast, the fungal isolate used to inoculate seeds explained only 8.4% of the variance (Fig. 4). These results suggest that under natural microbial soil conditions experienced by common bean farmers, seed inoculations with *B. bassiana* or *M. anisopliae* are unlikely to yield predictable levels of endophytic colonization.

## 4 Discussion

At least 62 peer-reviewed papers have been published on the inoculation of 30 plant species in 15 families with various fungal entomopathogens, e.g., *B. bassiana*, *Lecanicillium* sp., *L. lecanii*, *M. anisopliae*, *M. brunneum*, *M. robertsii*, and *Purpureocillium lilacinum* (Vega and Parsa, 2017). The plants studied the most are maize (10 papers), tomatoes (9 papers), and the common bean (9 papers).

The papers dealing with the common bean have examined seed dressing (Ownley et al., 2008) and seed soaking (Akutse et al., 2013, Mutune et al., 2016, Sasan and Bidochka, 2012), foliar sprays (Gurulingappa et al., 2010, Parsa et al., 2013), mycosed insects placed in the soil (Behie et al., 2012), soil drenching (Parsa et al., 2013), and fungal plugs placed in the soil (Behie et al., 2015, Sasan and Bidochka, 2013). In most of these common bean studies, colonization has been positive, with no endophytism when seeds were soaked with *M. robertsii* (Sasan and Bidochka, 2012) or *M. anisopliae* (Akutse et al., 2013, Mutune et al., 2016). In two studies, colonization by *M. robertsii* was assumed but not assessed experimentally (Behie et al., 2012, Sasan and Bidochka, 2013).

The medium used for growing common beans varied in the nine papers cited above, and with the exception of Sasan and Bidochka (2012), which included a non-sterile potting mixture, has always been sterilized: a “gnotobiotic” (i.e., sterile) medium not described (Ownley et al., 2008), sterile soil:sand, sterile soil:manure, or a sterile potting mixture (Akutse et al., 2013, Behie et al., 2012, Behie et al., 2015, Gurulingappa et al., 2010, Mutune et al., 2016, Parsa et al., 2013, Sasan and Bidochka, 2012). Our results indicate that under natural soil conditions experienced by common bean farmers (i.e., non-sterile soils), seed inoculations with *B. bassiana* or *M. anisopliae* are unlikely to yield predictable levels of endophytic colonization. As reported in Cherry’s et al. (2004) study using maize seed dressings with *B. bassiana* planted in the field, “Not all observed treatment effects were significant but field experiments had high sampling and residual errors which may, in some cases, have masked real differences, and any further studies will need greater sample sizes to minimize these errors.” We submit that this high sampling and residual error is what is to be expected under natural field conditions with non-sterile soil, in contrast to more controlled conditions when sterile substrates are used. Additionally, larger sample sizes would not “minimize these errors” since they are an inherent part of natural biological and spatial variation, though larger sample sizes would help more accurately characterize them.

Most endophyte seed-related experiments with other plants use sterile substrates, and some studies do not experimentally assess endophytism. Cherry et al. (2004) inoculated maize seeds with *B. bassiana* dry conidia (seed dressings) and planted the seeds in the field. Negative effects on *Sesamia calamistis* (Lepidoptera: Noctuidae), were ascribed to *B. bassiana*, even though no attempt was made to determine whether *B. bassiana* had become endophytic. Using maize seed (*Zea mays*) coated with *M. anisopliae* conidia, followed by field planting, Kabaluk and Ericsson (2007) reported positive effects on plant growth, but endophytism was not assessed. Working with tomatoes (*Solanum lycopersicum*), Powell et al. (2009) reported endophytism after seed coating with *B. bassiana* conidia and seed germination in sterile vermiculite, followed by replanting in sterile potting medium. Endophytic colonization by *B. bassiana* in sorghum following seed dressing, foliar or soil sprays was influenced by the substrate used, i.e., sterile soil, non-sterile soil, or vermiculite (Tefera and Vidal, 2009): “Seed inoculation with conidia caused no stem or leaf colonization by the fungus in non-sterile soil but did result in substantial endophytic colonization in vermiculite and sterile soil.” Quesada-Moraga et al. (2009) used *B. bassiana* for seed dressings of opium poppy (*Papaver somniferum*), which resulted in endophytism when seeds were germinated in sterile vermiculite and then transferred to sterile potting medium. Akello and Sikora (2012) soaked seeds of faba beans (*Vicia faba*) in conidial suspensions of *B. bassiana* or *M. anisopliae*, and planted the seeds in sterile soil:sand, with positive endophyte recovery. Akutse et al. (2013) soaked *V. faba* and common bean seeds in *B. bassiana* and *M. anisopliae* conidial suspensions, planting the seeds in a sterile soil:manure mixture. Endophytism was positive for *B. bassiana* and negative for *M. anisopliae*. Biswas et al. (2013) soaked seeds of white jute (*Corchorus capsularis*) in *B. bassiana* conidial suspensions, followed by planting in sterile soil, resulting in positive endophytism. Razinger et al. (2014) reported increased root and shoot fresh dry weight of cauliflower plantlets growing in non-sterile soil inoculated with *B. bassiana*. Castillo Lopez et al. (2014) soaked cotton seeds (*Gossypium hirsutum*) in conidial suspensions of *B. bassiana* or *Purpureocillium lilacinum*, with positive endophytism when seeds were grown in non-sterile potting medium; when seeds were directly planted in the field, endophytism was assumed, but not experimentally determined. Similarly, Castillo Lopez and Sword (2015) soaked cotton seeds in conidial suspensions of *B. bassiana* or *P. lilacinum* and planted seeds in non-sterile potting medium, where endophytism was assumed based on previous work, but not experimentally determined. Lohse et al. (2015) tested various formulations (encapsulation, film coating, sprays) of submerged conidiospore or blastospores of *B. bassiana* as inoculants for oilseed rape (*Brassica napus*) and the filmcoated seeds “was not evaluated further due to the suppressive effect of non-sterile soil observed with the encapsulated fungus.” Peña-Peña et al. (2015) soaked corn seeds in a conidial suspension of *Metarhizium pingshaense*, followed by planting in peat compost (authors don’t mention whether it was sterile or not), and reported positive endophytic recovery from roots.

The above papers reveal that there is a dearth of knowledge related to seed-related endophytic studies under natural conditions. Such studies are important because soil microbiota can have inhibitory effects on fungal entomopathogens (Scheepmaker and Butt, 2010). For example, Pereira et al. (1993) have shown antagonistic soil effects on *B. bassiana* when conidia are in contact with non-sterile soil, and these effects could be due to antagonistic soil microbial activity (Fargues et al., 1983, Lingg and Donaldson, 1981, Majchrowicz et al., 1990, Shields et al., 1981, Studdert and Kaya, 1990, Studdert et al., 1990). In a laboratory experiment, Kessler et al. (2003) reported higher colony forming units of *B. brongniartii* in three types of sterile soils than in non-sterile soils one month after incubation at two different temperatures and ascribed the higher levels to the “removal of antagonistic microbes” in sterile soil.

Thus, in terms of practical applications and implications, it is of paramount importance to consider whether the substrate used in endophyte-related research should be sterilized or not. The main reason for this is that it is important to consider whether the results will have external validity. As stated by Rosenheim et al. (2011), “narrowly controlled environmental conditions of experimental studies give strong “internal validity” but may restrict the ability to extend conclusions to situations of different environmental conditions (i.e., limited “external validity”). Even though studies that sterilize the substrate have merit and strong internal validity, they very likely will have limited external validity because sterile soils are not present in nature. Therefore, the practical implications of research using sterile substrates in seed-related experiments involving fungal entomopathogens as endophytes are questionable. Rosenheim et al. (2011) summarize the situation as follows: “… researchers often augment the statistical power of their experiments by holding environmental conditions as nearly constant as possible. Although this approach has obvious merits, it does raise the question of whether or not the conclusions derived from the experiment are relevant to farming operations that are conducted under other conditions (e.g., different crop cultivars, soil types, microclimates, or agronomic practices; presence of other members of a frequently speciose food web centered on the crop plant, including other herbivores, plant pathogens, omnivores, and predators).” Seeds are usually sterilized before inoculation with a fungal entomopathogen. One exception is a study by Keyser et al. (2014) that does not focus on endophytism. Wheat seeds were soaked with *M. brunneum* or *M. robertsii* and in order “… to maintain a realistic seed-borne microbial community the wheat seeds were not surface sterilized.” This approach leads towards increased external validity, and should be an important consideration in future studies.

A related problem is that if a study uses both sterile and non-sterile substrates, a rare occurrence in endophyte-related studies (Lohse et al., 2015, Razinger et al., 2014, Sasan and Bidochka, 2012, Tefera and Vidal, 2009), the process of sterilization will change the substrate properties, as has been widely reported in the literature (Berns et al., 2008, Egli et al., 2006, He and Cui, 2009, Lotrario et al., 1995, Perkins et al., 2013, Shaw et al., 1999, Urbanek et al., 2010). In addition, the sterilization process kills not only harmful organisms, but also beneficials; therefore, the microbial diversity in the sterile soil will not be the same as the non-sterile soil (Bennett et al., 2003, Katznelson, 1940, Trevors, 1996, Warcup, 1950). Consequently, a sterile substrate might not be comparable to the same substrate when it is non-sterile, in terms of biological, chemical and physical properties; therefore, it will not be clear what is causing the differences found in the study. When all these factors are considered, the best option would be to use non-sterile substrates, which mimics natural conditions but also increases the chances of non-repeatable results due to the inherent microbial diversity (within and between locations) and their antagonistic activity towards fungal entomopathogens.

Another issue to consider in seed-related experiments is the presence of seed-borne fungal endophytes. Parsa et al. (2016) identified 42 taxa of endophytic fungal endophytes in germinated seeds of 11 Colombian cultivars of the common bean. For that reason, in order for any targeted fungal entomopathogen to become endophytic after seed soaking, it will have to compete with seed-borne fungal endophytes. Based on the common bean results presented in the nine papers cited above as well as in the present paper, it is evident that *Metarhizium* species are not as successful as *B. bassiana* in their ability to become endophytic when seeds are soaked, possibly due to differential competition outcomes with seed-borne fungal endophytes, a research area in need of further study.

In conclusion, it is essential for research involving endophytic fungal entomopathogens to consider the conditions prevalent in the field, in order to increase external validity. Otherwise, there will be an abundance of studies that have strong internal validity and no chance of being transferred to the field. Individual testing of many different isolates of fungal entomopathogens might reveal some that are better able to become established as endophytes under non-sterile soil conditions (field conditions), which would then allow to investigate the mechanism responsible for improved establishment and hopefully improve our approach to their use as effective endophytic biological control agents. Simultaneous plant inoculations with different species or genera of fungal entomopathogens might also yield interesting findings. Finally, it is important to consider more in-depth statistical analyses such as a variance decomposition approach, which should aid in gaining a better understanding of the experimental results, rather than the usual focus on main treatment effects (mean responses).
